# EGCG Alleviates Obesity-Induced Myocardial Fibrosis in Rats by Enhancing Expression of SCN5A

**DOI:** 10.3389/fcvm.2022.869279

**Published:** 2022-04-29

**Authors:** Haoan Yi, Cong Liu, Jing Shi, Shuo Wang, Haoxin Zhang, Yongshu He, Jianping Tao, Shude Li, Renfa Zhang

**Affiliations:** ^1^Department of Cell Biology and Medical Genetics, School of Basic Medicine, Kunming Medical University, Kunming, China; ^2^Department of Anesthesiology, The Second Affiliated Hospital of Kunming Medical University, Kunming, China; ^3^Department of Orthopedics, Tangdu Hospital, The Fourth Military Medical University, Xi'an, China; ^4^Department of Biochemistry and Molecular Biology, School of Basic Medicine, Kunming Medical University, Kunming, China; ^5^Department of Pharmacology, School of Basic Medicine, Kunming Medical University, Kunming, China; ^6^Department of Orthopaedics, The Second Affiliated Hospital of Kunming Medical University, Kunming, China; ^7^Pharmaceutical Science and Yunnan Key Laboratory of Pharmacology for Natural Products, Kunming Medical University, Kunming, China; ^8^Department of Physical Education, Kunming Medical University, Kunming, China

**Keywords:** EGCG, myocardial fibrosis, SCN5A, obesity, RNA-Seq

## Abstract

**Object:**

Obesity is an increase in body weight beyond the limitation of skeletal and physical requirement, as the result of an excessive accumulation of fat in the body. Obesity could increase the risk of myocardial fibrosis. (-)-Epigallocatechin-3-gallate (EGCG) is the most abundant substance in green tea and has been reported to have multiple pharmacological activities. However, there is not enough evidence to show that EGCG has a therapeutic effect on obesity-induced myocardial fibrosis. This study aims to investigate whether EGCG is a potential drug for obesity-induced myocardial fibrosis.

**Methods:**

Obesity-induced myocardial fibrosis rat model was established by HFD feeding for 36 weeks. EGCG was intragastrically administered at 160 mg/kg/d for the last 4 weeks. The pathological changes of myocardial fibrosis were evaluated by tissue pathological staining and collagen quantification. Furthermore, total RNA was extracted from the heart for RNA-seq to identify the changes in the transcript profile, and the relevant hub genes were verified by quantitative real-time PCR and western blot.

**Results:**

EGCG significantly relieved HFD diet-induced obesity and alleviated the pathology of myocardial fibrosis. Biochemical analysis showed that EGCG could relieve the burden of lipid metabolism and injury to the myocardium and transcript profile analysis showed that EGCG could alleviate obesity-induced myocardial fibrosis by increasing the level of Scn5a in the heart. Furthermore, quantitative real-time PCR and western blot analysis for SCN5A also confirmed this finding.

**Conclusion:**

Taken together, these results suggest that EGCG could protect against the obesity-induced myocardial fibrosis. EGCG plays an anti-myocardial fibrosis role by regulating the expression of SCN5A in the heart.

## Introduction

**Figure 1:**
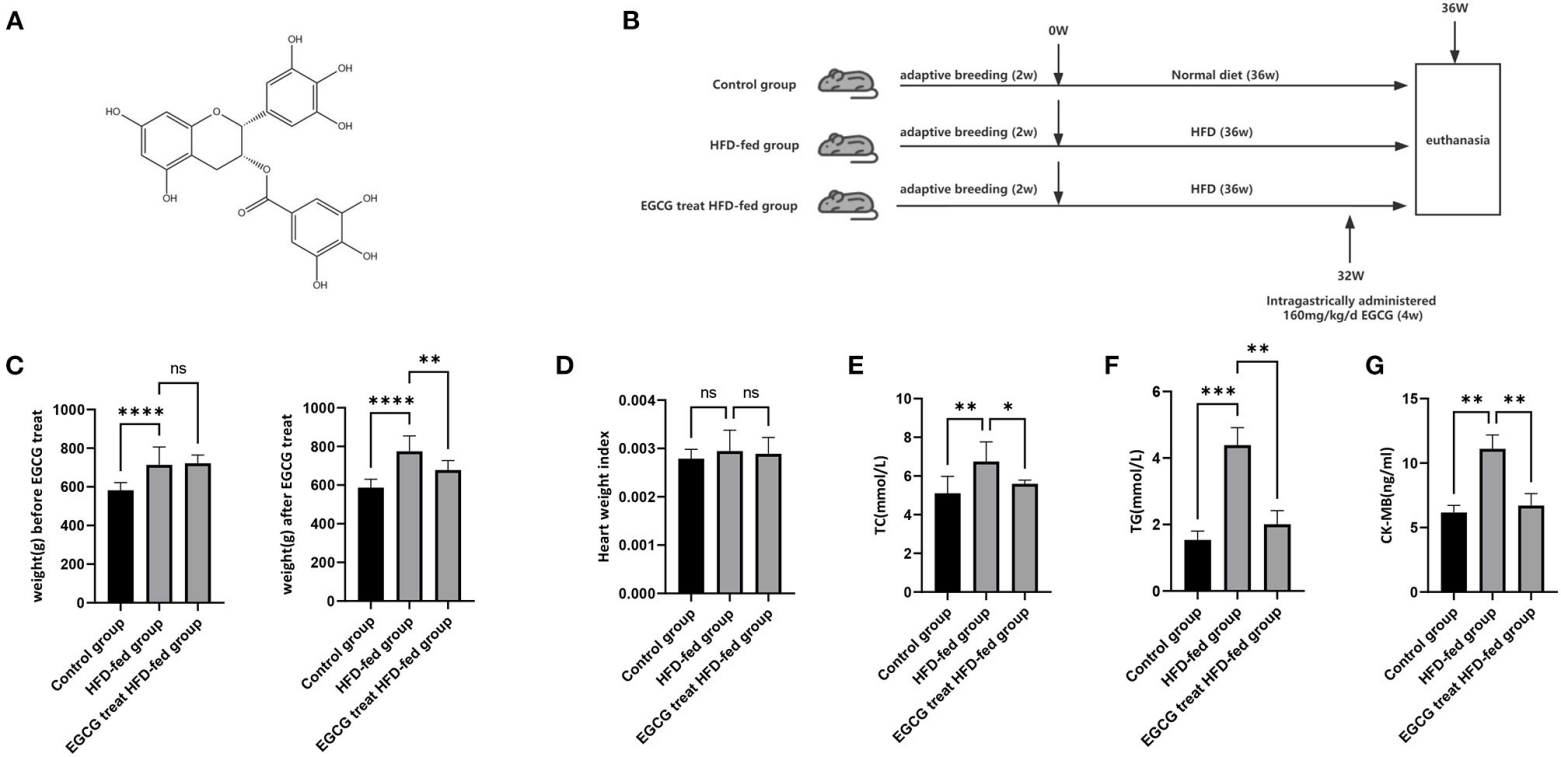
EGCG could relieve the burden of lipid metabolism and myocardium injury. **(A)** Chemical structure of EGCG; **(B)** Schematic diagram of the experimental setup of HFD-fed and EGCG treatment; **(C)** The effect of HFD and EGCG treatment on weight; **(D)** The effect of HFD and EGCG treatment on heart weight index. The heart weight index was calculated as HWI = heart weight (HW)/body weight (BW); **(E)** The effect of HFD and EGCG treatment on serum TC levels was quantified; **(F)** The effect of HFD and EGCG treatment on serum TG levels was quantified; **(G)** The effect of HFD and EGCG treatment on serum CK-MB levels was quantified. (*p < 0.05, **p < 0.01, ***p < 0.001).

Over the last decade, obesity has become increasingly common around the world (1, 2). Although we have now a deeper understanding of obesity, it must be admitted that obesity still has been threatening our life and health increasingly. Obesity is a major risk factor for various chronic diseases, including the cardiovascular diseases. Obesity could cause heart dysfunction via disturbance of the metabolism and function of cardiomyocytes (3). Furthermore, diabetes and hypertension due to obesity also increase the risk of cardiovascular diseases (4, 5). The intake of a high-fat diet (HFD) could increase the burden of the heart muscle and lead to myocardial fibrosis (6). Myocardial fibrosis is characterized by net accumulation of extracellular matrix (ECM) proteins, which is caused by the frequent repair of myocardium damage (7). Several drugs have been shown to be effective in treating myocardial fibrosis (8–11). However, more drugs with potential to prevent or treat obesity-induced myocardial fibrosis need to be discovered.

(-)-Epigallocatechin-3-gallate (EGCG) is the most abundant substance in green tea (12) (Figure 1A) and has been reported to have many pharmacological activities and remarkable efficacy in the treatment of many diseases (13). EGCG can improve the chronic alcohol-induced cognitive dysfunction by interfering with neuroinflammation, cell death and the oxido-nitrosative cascade (14) and has a protective effect against NAFLD through the regulation of oxidative stress (15). The latest studies suggest that EGCG may be a potential drug for COVID-19 (16). However, it is unknown whether EGCG could be a potential drug for obesity-induced myocardial fibrosis.

SCN5A is a carrier of Na^+^ ion channels located on the cell surface, which is essential for the cardiac potential regulation (17). Mutations in the SCN5A gene may increase the risk of certain heart diseases (18). Additionally, Scn5a plays an important role in the process of age-related myocardial fibrosis (19). The bioinformatics result in this study shows that there is a close target relationship between EGCG and Scn5a, suggesting that EGCG may alleviate obesity-induced myocardial fibrosis by regulating the expression of Scn5a.

In this context, the aim of this study was to investigate whether EGCG could be a potential drug for obesity-induced myocardial fibrosis. HFD feeding was used to establish an obesity model and EGCG intervention was used in rats to evaluate the preventative or therapeutic effect of EGCG on obesity-induced myocardial fibrosis. Furthermore, RNA-seq was conducted to explorer and verify that EGCG could alleviate obesity-induced myocardial fibrosis by regulating the expression of Scn5a.

## Materials and Methods

### Animals and Treatment

EGCG (>98% purity) was obtained from School of Pharmaceutical Science, Kunming Medical University. Male Sprague–Dawley (SD) rats (SPF, 6 weeks old, 200 ± 20 g) were obtained from the Animal Research Center of Kunming Medical University. Animals were maintained at a controlled temperature (24 ± 2°C) and humidity (60 ± 10%) with a 12 h light/12 h dark cycle. After two weeks of adaptive breeding, SD rats were randomly divided into three groups: a) the control group (*n* = 10), with a normal diet for 36 weeks; b) HFD-fed group (*n* = 10), with the HFD (15% lard, 30% sucrose, 2% cholesterol, 1% sodium cholate, 5% protein powder, and 47% regular diet), in which sucrose can be converted into energy and fat, for 36 weeks; c) EGCG-treated HFD-fed group (*n* = 10), with the HFD for 36 weeks and intragastrically administered with 160 mg/kg/d EGCG for the last 4 weeks (Figure 1B). According to previous studies, 100–200 mg/kg/d EGCG treatment could provide therapeutic effects on early aged hypertension-induced neural apoptosis (20) and senescence-mediated redox imbalance in the livers and kidneys in mice (21). Therefore, we chose 160 mg/kg/d as our experimental dose in our study. This study was approved by the Institutional Review Boards of Kunming Medical University.

### Biochemical Assay

Peripheral blood samples were centrifuged at 4,000 g for 10 min at 4°C, and the cleared supernatants were collected. Total cholesterol (TC) and triglycerides (TG) in the serum were determined by using a commercial kit (Leagene Biotechnology, Beijing, China). The concentration of creatine phosphokinase-MB (CKMB) in the serum was determined by an ELISA kit (Xinyu Biotechnology, Shanghai, China). All operations were carried out according to the manufacturer's instructions.

### Pathological Staining

A segment of the heart tissue was excised, fixed in 4% paraformaldehyde for 24 h and embedded in paraffin. Serial 5-μm-thick sections were mounted and stained with hematoxylin and eosin (HE) and Masson's trichrome to examine the pathology of the heart. Furthermore, immune histology staining was performed for collagen I (1:200, AF7001, Affinity Biosciences, USA) and collagen III (1:200, AF0136, Affinity Biosciences, USA) in heart sections. Quantitative assessment of the fibrosis was determined based upon the extent of patchy and interstitial fibrosis. Each heart was observed, and at least 5 pictures were obtained in each region.

### RNA Extraction and Quantitative Real-Time PCR

Total RNA was extracted from the hearts with RNAiso plus (TaKaRa Biotechnology, Beijing, China). The mRNA was reverse transcribed into cDNA using a RevertAid First Strand cDNA Synthesis Kit (Thermo Scientific, USA). Quantitative real-time PCR was performed using SYBR green PCR master mix (Roche, Penzberg, Germany) for 40 cycles in an ABI Q6 apparatus (Applied Biosystems, CA, USA). Real-time fluorescence quantitative PCR was performed using the following primers: Gapdh forwards primer, 5′-TGGCACCACACCTTCTACAATG-3′; Gapdh reverse primer, 5′-TCATCTTCTCGCGGTTGGC-3′; Scn5a forwards primer, 5′-GAAGTGCCAGCGTAACTCCTA-3′; and Scn5a reverse primer, 5′-TACCTCAAACACCCCATTCAC-3′.

### RNA-Seq and Bioinformation Analysis

Three hearts from each group were subjected to RNA-seq. RNA libraries were generated using Illumina's TruSeq Stranded Total RNA Sample Prep Kit. RNA libraries were sequenced using paired-end 150 bp reads on a NovaSeq system (Illumina, San Diego, CA, USA). Raw read data were subjected to the quality control using FastQC, and unqualified reads were cleaned using fastp (22). The filtered read data were aligned to the Rnor6.0 genome (release 100 in Ensemble) by using HISAT2 (23). Differential expression analysis was performed using DESeq2 (24) and the protein–protein interaction (PPI) network was predicted using the STRING online database (http://string-db.org). Firstly, the gene list was typed into the database. Then, PPI was constructed with the default parameters and displayed with CytoScape (25). Further, the hub genes in the PPI networks were identified using Cytohubba (26), a plugin in Cytoscape software, and the top nodes were ranked by degree.

### Western Blot

Total protein from the heart was extracted with ice-cold 1% Triton X-100 RIPA lysate supplemented with PMSF and then quantified by BCA. The protein was loaded in lanes, separated by SDS–PAGE and transferred to a polyvinylidene fluoride (PVDF) membrane. The PVDF membrane was incubated with collagen I (1:1000, AF7001, Affinity Biosciences, USA), collagen III (1:1000, AF0136, Affinity Biosciences, USA), Scn5a antibody (1:1000, AF0255, Affinity Biosciences, USA) and GAPDH antibody (1:1000, D16H11, Cell Signaling Technology, USA) overnight at 4°C. Then, the membrane was incubated with HRP-labeled antibodies and exposed to HRP chemiluminescence. ImageJ 1.52 was used to quantify the bands, and GAPDH was used as a loading control to calculate the relative quantification among each group.

### Statistical Analysis

Data were presented as the mean and standard error of the mean (mean ± SEM). The Kolmogorov–Smirnov test showed that the data were normally distributed. Differences were evaluated by one-way ANOVA for multigroup comparisons and SNK-Q test for pairwise comparisons. Statistical analysis was performed using SPSS 21.0 and GraphPad Prism 8.0 statistical software. Statistical significance level was set at α = 0.05.

## Results

### EGCG Could Relieve the Burden of the Lipid Metabolism and Myocardium Injury

After 32 weeks of HFD and normal diet, the weight of HFD-fed group was significantly higher than that of the control group. (Further, compared with the HFD-fed group, the weight of the EGCG-treated HFD-fed group decreased significantly after 4 weeks of EGCG treatment (*P* < 0.0001, One-way ANOVA and SNK-Q test, Figure 1C). These results showed that HFD-feeding significantly induced the obesity and EGCG treatment could significantly reduce the obesity in rats. At this point, the rats were euthanized for further experimental evaluation. Heart weight index was an indicator of cardiac hypertrophy measured by heart weight to body weight (HW/BW) ratio. However, no change was found in the heart weight index (*P* > 0.05, one-way ANOVA test, Figure 1D). Furthermore, the TC and TG levels in the serum were examined and the results showed that HFD feeding could increase the level of blood lipids, while EGCG treatment could significantly decrease the HFD feeding-induced increase in the level of blood lipids (*P* < 0.0001, one-way ANOVA and SNK-Q test, Figures 1E,F). This result indicated that EGCG could reduce obesity-induced discordance of the lipid metabolism. Discord lipid metabolism could place a severe burden on the heart, so we measured CKMB in the serum to assess the myocardial injury and found that serum CKMB concentration was highest in the HFD-Fed group and lowest in the control group (*P* < 0.0001, one-way ANOVA and SNK-Q test, Figure 1G). The results showed that HFD feeding aggravated the myocardial injury and EGCG effectively protected against obesity-induced myocardial injury.

### EGCG Alleviated the Pathology of Obesity-Induced Myocardial Fibrosis

**Figure 2:**
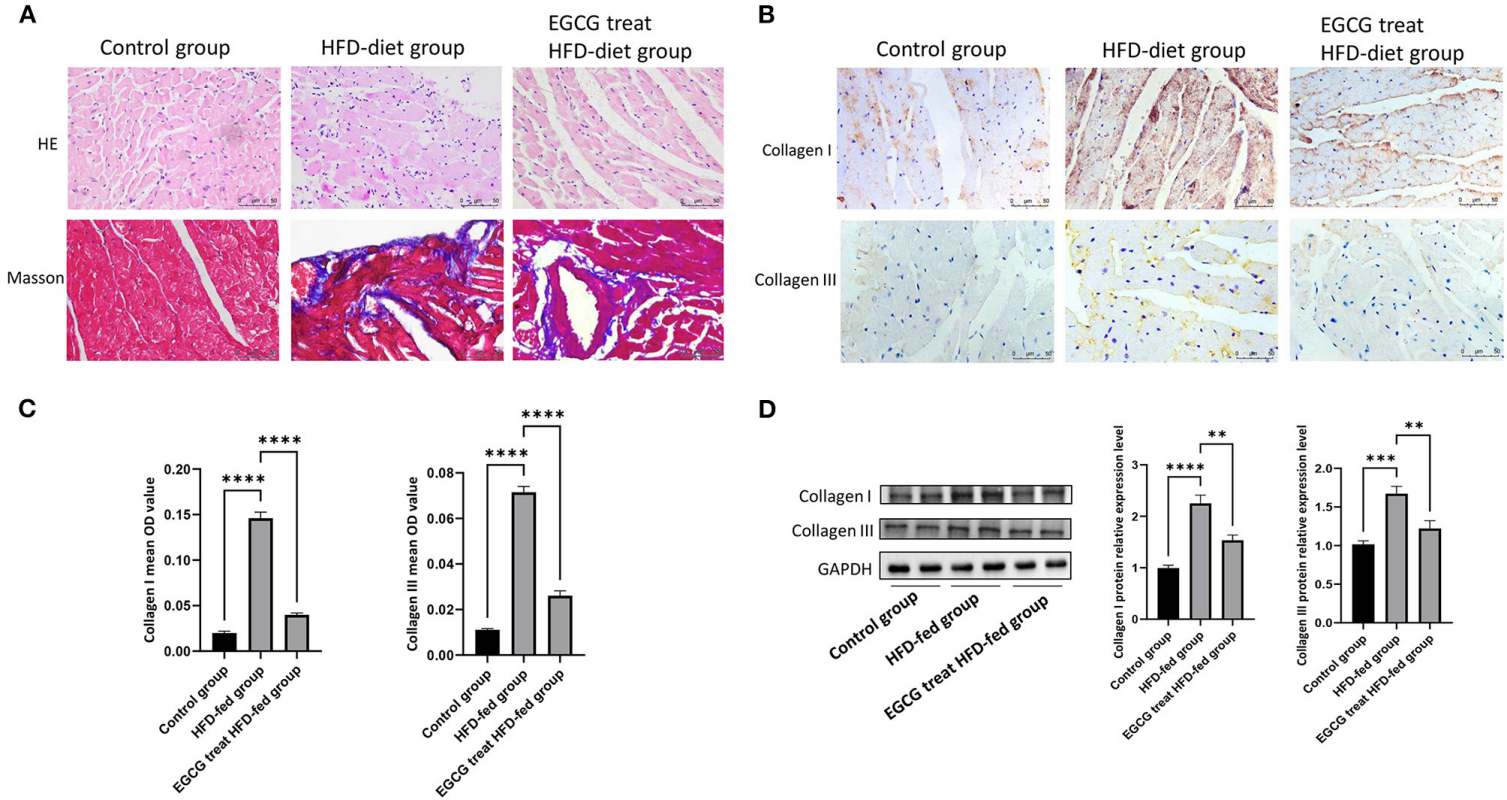
EGCG alleviated the pathology of obesity-induced myocardial fibrosis. **(A)** Effect of EGCG on obesity-induced myocardial fibrosis in heart tissue by HE and Masson staining; **(B)** Effect of EGCG on the deposition of collagen I and collagen III in heart tissue by Immunohistochemical staining; **(C)** The mean OD values of collagen I and collagen III in heart tissue by Immunohistochemical staining; **(D)** Effect of EGCG on the expression of collagen I and collagen III proteins were detected by western blot with GAPDH as a loading control in the heart. (*p < 0.05, **p < 0.01, ***p < 0.001).

Frequent injury and repair of the myocardium can lead to the formation of fibrosis. To investigate the effect of EGCG on the pathological changes in HFD-fed rats, heart tissues were stained with HE and Masson's trichrome separately. HE staining results showed that the tissues in the control group were evenly distributed without obvious inflammatory cell infiltration, while tissues in the HFD-fed group revealed pyknosis, blurred cell boundaries, inflammatory cell infiltration and connective tissue hyperplasia. After EGCG treatment, the infiltration of inflammatory cells was significantly reduced, nuclear pyknosis and connective tissue hyperplasia were significantly improved, the pathological change was rescued. The Masson staining results confirmed the presence of extensive myocardial fibrosis in the HFD-fed group compared to the control group, while the level of myocardial fibrosis in the EGCG-treated HFD-fed group was significantly reduced (Figure 2A).

Furthermore, the accumulation levels of collagen I and collagen III in hearts were analyzed. As shown by immunohistochemistry and western blot analysis, HFD can significantly induce the expression and obvious deposition of collagen I and collagen III in ECM. However, deposition (*P* < 0.0001, one-way ANOVA and SNK-Q test, Figures 2B,C) and expression (*P* < 0.0001, one-way ANOVA and SNK-Q test, Figure 2D) of collagen I and collagen III were significantly inhibited by EGCG treatment in HFD-fed rats.

### RNA-Seq Reflected the Different Gene Expression Profiles in the Heart

**Figure 3:**
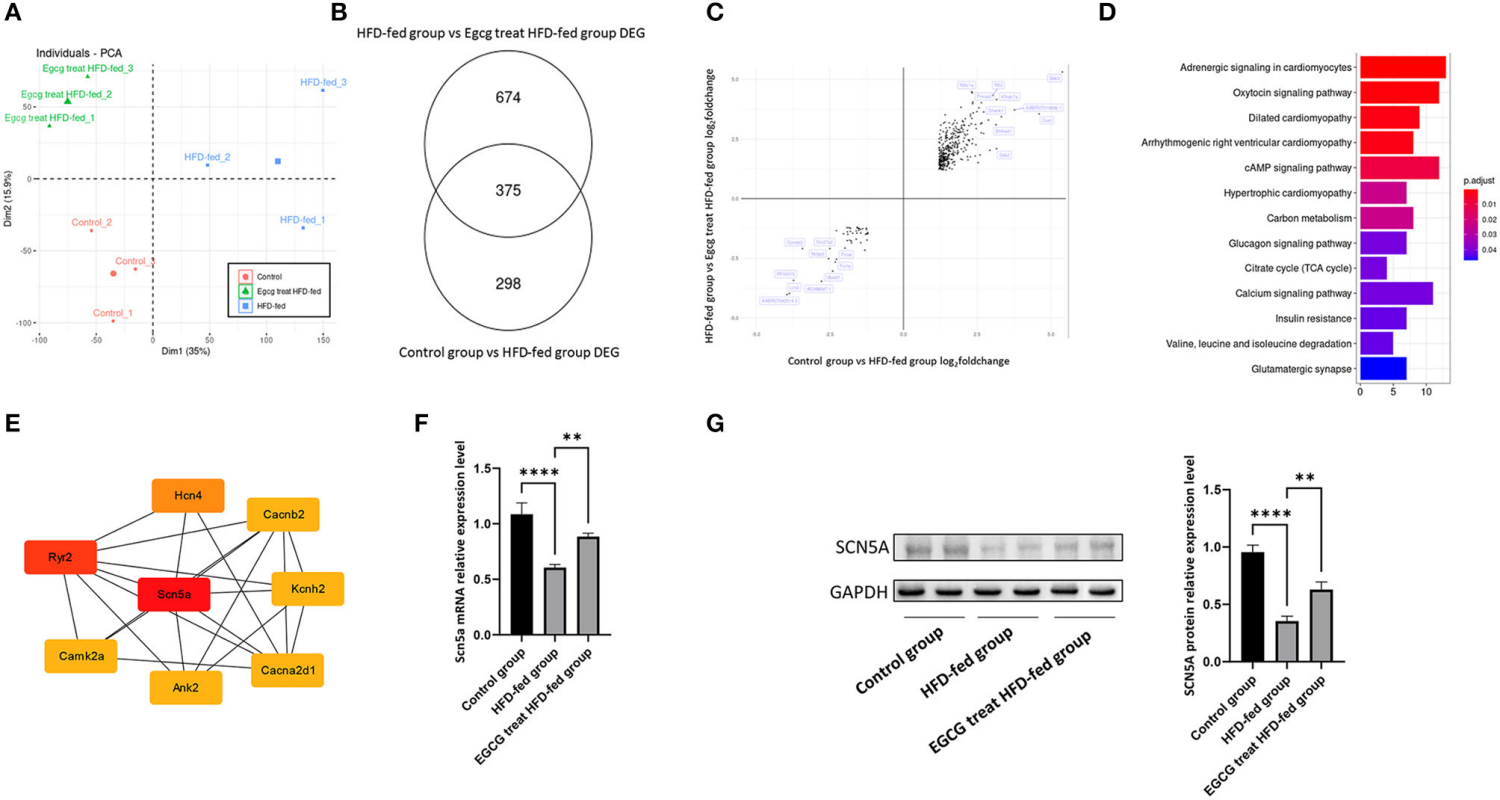
RNA-seq reflect Scn5a is a hub gene of EGCG in alleviating obesity-induced myocardial fibrosis. **(A)** Principal Component Analysis was performed on RNA-seq; **(B)** The number of differently expression gene was identified by Deseq2; **(C)** The cross-volcano map reflects the reverse of gene expression by EGCG treatment; **(D)** The KEGG pathway enrichment pf reversed gene sets revealed that the change of glucose and lipid metabolism; **(E)** PPI network analysis showed that Scn5a was a hub gene in EGCG alleviating obesity-induced myocardial fibrosis; **(F)** The relative mRNA expression levels of Scn5a in the different groups; **(G)** The effect of EGCG on the expression of SCN5A proteins was detected by western blot with GAPDH as a loading control in the heart. (*p < 0.05, **p < 0.01, ***p < 0.001).

RNA-seq was performed to explore the potential mechanism of EGCG in the treatment of obesity-induced myocardial fibrosis. The read counts were normalized by using DESeq2 before analysis. The principal component analysis (PCA) of the transcriptome data was performed first. The first principal component from this analysis accounted for 35% of total transcriptome variance, it completely separated the control group from other groups. The second principal component from this analysis accounted for 15.9% of total transcriptome variance, it completely separated the HFD-fed group from other groups. The result from PCA revealed the different gene expression value induced by HFD-fed and the treatment of EGCG (Figure 3A). The PCA results revealed the different gene expression profiles induced by HFD feeding and EGCG treatment in hearts.

Then, we identified differentially expressed genes by DESeq2 based on strict statistical criteria (log_2_Fold_change > 1.2 and adjusted *P* value < 0.05). A total of 673 differentially expressed genes, including 275 upregulated and 398 downregulated genes, were identified in the comparison of the control group and HFD-fed group (Supplementary Table 1). A total of 1049 differentially expressed genes, including 738 upregulated genes and 311 downregulated genes, were identified in comparison with the EGCG-treated HFD-fed group and the HFD-fed group (Supplementary Table 2, Figure 3B). This result indicated that both HFD-fed and EGCG treatment resulted in the significant changes in the transcriptome.

To explore the mechanism by which EGCG alleviates obesity-induced myocardial fibrosis, some genes that were changed by a HFD diet and reversed by EGCG treatment were selected for further analysis. First, the intersection of the two analyses of differentially expressed genes was taken to establish a new gene set (Figure 3B). Then, the change direction of the genes in this new gene set, including 375 genes was analyzed. Interestingly, it was found that all the gene expression changes caused by the HFD diet could be reversed by EGCG treatment (Figure 3C). In the end, a total of 375 genes were included in the further analysis. To understand the function of these genes, we enriched these genes with the KEGG database and found that these genes were mainly involved in the pathways related to the heart disease, glycolipid metabolism and calcium signaling, suggesting that EGCG treatment could alleviate the obesity-induced cardiac pathological changes via redistribution of ions or equilibrium of glycolipid metabolism (Figure 3D). A PPI network of these genes was constructed and the core of the network was found to be the sodium-calcium channel, including Scn5a, Ryr2, Hcn4, Cacna2d1, Camk2a, Ank2, Kcnh2 and Cacnb2. The Scn5a gene had the highest degree score of 26 (Figure 3E). Therefore, the expression of Scn5a gene may play a central role in EGCG in alleviating HFD-fed induced myocardial fibrosis.

### Scn5a Was a Hub Gene of EGCG in Alleviating Myocardial Fibrosis

The above bioinformatics analysis showed that EGCG could alleviate obesity-induced myocardial fibrosis by regulating the expression of Scn5a. Previous studies showed that Scn5a knockout in mice accelerates the progression of myocardial fibrosis (19). The quantitative real-time PCR and western blotting conducted with SCN5A showed that HFD-fed could significantly reduce the expression level of SCN5A, which may be the main reason for the occurrence of myocardial fibrosis. While EGCG treatment could significantly increase the expression of SCN5A both at mRNA level and protein level, it could also reverse the decreased expression level of SCN5A induced by HFD-Fed (*P* < 0.0001, one-way ANOVA and SNK-Q test, Figures 3F,G).

## Discussion

Obesity is gradually becoming a public health concern (27). Long-term unhealthy diets could increase the risk of obesity and lead to the fibrotic changes in the heart muscle (28). Myocardial fibrosis is defined as the imbalance of ECM production and degradation and is a common pathological alteration during the development of various heart diseases (29). Myocardial fibrosis is the primary manifestation of cardiac remodeling (30, 31) and a predictive factor for sudden cardiac death (32). However, drugs that could prevent obesity-induced myocardial fibrosis are rare. EGCG, as the main abundant substance in green tea, has been found to be effective in treating a variety of diseases and has antioxidative, anti-inflammatory and anticancer properties (33). Therefore, this study aims to investigate the effect of EGCG on the treatment of obesity-induced myocardial fibrosis.

In this study, we established the obesity models by feeding rats with a HFD. After several weeks of feeding, the weight of the rats in the HFD-fed group increased significantly compared to that of the rats in the control group. Furthermore, we found that the rats in the EGCG-treated HFD-fed group lost weight, reduced glucose and lipid metabolism disorders and reduced myocardial injury after EGCG treatment. The myocardial collagen network was mainly composed of collagen I and collagen III, which provided a supportive framework for cardiomyocytes and determine ventricular compliance (34). The results revealed that the levels of myocardial fibrosis and collagen expression were significantly reduced after EGCG treatment. It is worth noting that high doses (500 mg/kg/d) of EGCG treatment could induce myocardial fibrosis in mice (35). However, we found that 160 mg/kg/d doses of EGCG treatment alleviated obesity-induced myocardial fibrosis but did not find that it could aggravate the manifestations of obesity-induced myocardial fibrosis in our study. This suggests that EGCG has a two-sided effect on obesity-induced myocardial fibrosis. In order to explore the mechanism of EGCG against obesity-induced myocardial fibrosis, we performed RNA-seq on rat heart tissue. After bioinformatic analysis, we found that the heart tissues from the control group, HFD-fed group and EGCG-treated HFD-fed group had completely the different gene expression profiles and that Scn5a played an important role in EGCG resistance to obesity-induced myocardial fibrosis. Our experimental results of Scn5a expression level were consistent with the transcriptomic profiles. Coincidentally, Scn5a was found to play a protective role in myocardial fibrosis.

Over the last few decades, an increasing number of human SCN5A gene mutations have been described, and these genetic variations have been reported to be associated with many different types of cardiac disorders, such as Brugada syndrome (36, 37), long QT syndrome (38), and cardiac conduction system dysfunction (39). These genotype and phenotype associations suggest that SCN5A is critical for the maintenance of normal cardiac physiological functions. However, the specific physiological mechanism of SCN5A-related cardiac diseases has not been fully elucidated due to the influence of genetic background and environmental factors on disease manifestations. The SCN5A gene encodes the alpha subunit of the main cardiac Na^+^ ion channel Nav1.5, which plays a critical role in the regulation of cardiac electrophysiological function (17). It is becoming increasingly clear that the function and regulation of Na^+^ ion channels are more complex than previously thought and that Na^+^ ion channels may play other roles that are unrecognized in the structure and function of the heart. Suppression of Scn5a gene expression could inhibit cardiac Na^+^ ion channels through Wnt signaling, lead to an increase in the QRS duration of electrocardiograms and eventually increase the susceptibility to ventricular tachycardia (40).

Moreover, our PPI analysis has found that Scn5a plays a core role, but most of the genes closely associated with it are associated with Ca^2+^ ion channels, such as Camk2a, Cacnb2 and Cacna2d1. KEGG enrichment of the differentially expressed genes also confirms this point. The distribution of these ions in the heart is critical for the maintenance of the normal physiological functions of the heart. Obesity could lead to a chaotic distribution of ions in the heart, and EGCG can rescue this abnormal distribution. The results of our study further confirm Feng et al.'s conclusion that EGCG can alleviate the heart failure by changing sarcoplasmic reticulum Ca^2+^ content, inhibiting Na^+^-Ca^2+^ exchange, regulating sarcoplasmic reticulum Ca^2+^ load, and regulating cardiomyocyte contraction (41). These findings support that EGCG can alleviate obesity-induced myocardial fibrosis by regulating Na^+^-Ca^2+^ ion channels. EGCG can significantly increase the expression of Na^+^-Ca^2+^ ion channel protein reduced by HFD feeding, thereby alleviating obesity-induced myocardial fibrosis.

Myocardial collagen depends on a dynamic balance between the synthesis and degradation, but if the balance is disrupted, this could cause collagen to accumulate gradually under the pathological conditions. The accumulation of collagen I and collagen III in the myocardium is the main culprit leading to myocardial fibrosis. SCN5A heterozygous knockout (Scn5a+/–) in mice could accelerate the process of myocardial fibrosis (19). These findings suggest that SCN5A could change the dynamic balance of myocardial collagen I and collagen III. Our western blot quantitative results for collagen I, collagen III and SCN5A also support this hypothesis. Therefore, the further problem worthy of our attention is how EGCG regulates the expression of SCN5A and then changes the accumulation of collagen to prevent obesity-induced myocardial fibrosis.

In conclusion, this study demonstrates that EGCG is a potential drug to relieve the obesity-induced myocardial fibrosis by regulating the expression of the Scn5a gene.

## Data Availability Statement

The original contributions presented in the study are publicly available. This data can be found here: SRA database, accession PRJNA810762.

## Ethics Statement

The animal study was reviewed and approved by the Institutional Review Boards of Kunming Medical University.

## Author Contributions

SL, RZ, and JT conceived, review & editing the study, and critically revising the manuscript. CL, JS, SW, and HZ conducted the experiments. HY, CL, JS, SW, and YH collected the data, processed data analysis, and interpretation of data. HY wrote the original manuscript. All authors have reviewed and approved the final manuscript.

## Funding

This work was supported by grants from the Scientific and Technological Development Project of Yunnan Province (2019FE001-189). This work was also supported by the Pharmaceutical Science & Yunnan Key Laboratory of Pharmacology for Natural Products.

## Conflict of Interest

The authors declare that the research was conducted in the absence of any commercial or financial relationships that could be construed as a potential conflict of interest.

## Publisher's Note

All claims expressed in this article are solely those of the authors and do not necessarily represent those of their affiliated organizations, or those of the publisher, the editors and the reviewers. Any product that may be evaluated in this article, or claim that may be made by its manufacturer, is not guaranteed or endorsed by the publisher.
